# Differentiation of pilocytic astrocytoma, medulloblastoma, and hemangioblastoma on diffusion-weighted and dynamic susceptibility contrast perfusion MRI

**DOI:** 10.1097/MD.0000000000031708

**Published:** 2022-11-04

**Authors:** Ryo Kurokawa, Mariko Kurokawa, Akira Baba, John Kim, Aristides Capizzano, Jayapalli Bapuraj, Ashok Srinivasan, Toshio Moritani

**Affiliations:** a Division of Neuroradiology, Department of Radiology, University of Michigan, Ann Arbor, Michigan, USA.

**Keywords:** dynamic susceptibility contrast, hemangioblastoma, magnetic resonance imaging, medulloblastoma, pilocytic astrocytoma

## Abstract

This study aimed to evaluate the diagnostic performance of dynamic susceptibility contrast (DSC) perfusion magnetic resonance imaging and apparent diffusion coefficient (ADC) for differentiating common posterior fossa tumors, pilocytic astrocytoma (PA), medulloblastoma (MB), and hemangioblastoma (HB). Between January 2016 and April 2022, we enrolled 23 (median age, 7 years [range, 2–26]; 12 female), 13 (10 years [1–24]; 3 female), and 12 (43 years [23–73]; 7 female) patients with PA, MB, and HB, respectively. Normalized relative cerebral blood volume and flow (nrCBV and nrCBF) and normalized mean ADC (nADCmean) were calculated from volume-of-interest and statistically compared. nADCmean was significantly higher in PA than in MB (PA: median, 2.2 [range, 1.59–2.65] vs MB: 0.93 [0.70–1.37], *P* < .001). nrCBF was significantly higher in HB than in PA and MB (PA: 1.10 [0.54–2.26] vs MB: 1.62 [0.93–3.16] vs HB: 7.83 [2.75–20.1], all *P* < .001). nrCBV was significantly different between all 3 tumor types (PA: 0.89 [0.34–2.28] vs MB: 1.69 [0.93–4.23] vs HB: 8.48 [4.59–16.3], *P* = .008 for PA vs MB; *P* < .001 for PA vs HB and MB vs HB). All tumors were successfully differentiated using an algorithmic approach with a threshold value of 4.58 for nrCBV and subsequent threshold value of 1.38 for nADCmean. DSC parameters and nADCmean were significantly different between PA, MB, and HB. An algorithmic approach combining nrCBV and nADCmean may be useful for differentiating these tumor types.

## 1. Introduction

Pilocytic astrocytomas (PAs) and hemangioblastomas (HBs) are primary brain tumors that most frequently develop in the cerebellum.^[1,2]^ Both tumor types are potentially curable by surgical resection and are classified as central nervous system (CNS) World Health Organization (WHO) grade 1 tumors in the WHO CNS5.^[3]^ PAs and HBs are predominantly observed as solid and cystic tumors on imaging and may resemble each other but require distinct clinical and surgical approaches. HBs are highly vascularized tumors with large networks of tightly packed capillary vessels; thus, the entire tumor capsule should be removed during surgical resection to avoid major bleeding.^[4]^ Preoperative embolization is often performed to reduce tumor blood supply and avoid profuse intraoperative bleeding.^[4]^ In contrast, PAs are less vascularized tumors characterized by a biphasic pattern comprising densely populated components of differentiated pilocytes with bipolar processes and various degrees of loosely textured components with abundant myxoid stroma.^[1]^ Medulloblastomas (MBs) are CNS WHO grade 4 tumors that predominantly occur in the posterior fossa in children. Nevertheless, the incidence in adults is increasing, with a rate of approximately 0.5 adult patients per million per year and an oldest reported age of 88 years.^[5,6]^ PAs, MBs, and HBs may exhibit a similar appearance to each other in a similar location (i.e., posterior fossa) on conventional magnetic resonance imaging (MRI), but have divergent natural courses and management requirements.^[2,7]^ Therefore, high accuracy differentiation on preoperative MRI may contribute to clinical practice.

The diagnostic performance of advanced MRI sequences for differentiating these tumor types has been investigated. Most studies comparing PA and MB have evaluated diffusion MRI techniques, including apparent diffusion coefficient (ADC) values, histograms, and diffusion tensor imaging.^[1,3–15]^ A recent study reported that dynamic susceptibility contrast (DSC) perfusion MRI was useful for differentiating PA and MB.^[12]^ Few studies have reported the differential performance of DSC-MRI for PA and HB,^[2,16]^ and reports on the utility of ADC have often been negative.^[16,17]^

Although previous studies have predominantly focused on comparisons of 2 tumor types, the importance of multi-group comparisons is increasingly recognized due to increased clinical applicability and potential contribution to determine the initial decision model when developing a deep learning model for diagnostics.^[18–20]^ Nevertheless, there is a lack of multi-group comparisons of PA, MB, and HB using DSC-MRI or ADC due to potential bias and an insufficient number of cases. Therefore, this study aimed to evaluate the diagnostic performance of DSC-MRI and ADC in addition to the conventional MRI findings for differentiating PA, MB, and HB.

## 2. Materials and methods

### 2.1. Ethics

The study received approval from the institutional review board, and the need for consent was exempted due to the retrospective and noninvasive nature of the study. Data were acquired in compliance with all applicable Health Insurance Portability and Accountability Act regulations. Data were de-identified prior to analysis.

### 2.2. Patients

We searched the electronic database of our hospital for PA, MB, and HB between January 2016 and April 2022. Inclusion criteria were as follows: pathologically proven tumors and that pretreatment MRI including T2-weighted imaging or fluid-attenuated inversion recovery (FLAIR) imaging, pre-and post-contrast enhanced T1-weighted imaging, diffusion-weighted imaging (DWI) and DSC-MRI were performed. Twenty-six, thirteen, and thirteen patients with PA, MB, and HB, respectively, met the inclusion criteria. After excluding 3 and 1 patients with PA and HB, respectively, due to severe artifacts on DSC-MRI, a total of 48 patients (comprising 23, 13, and 12 patients with PA, MB, and HB, respectively) were finally included in the study for further analysis. Patients with PA and MB included patients who were investigated in our previous study.^[12]^

### 2.3. MRI protocols

Patients underwent brain MRI examinations in the supine position using 1.5 T (n = 34) and 3 T (n = 14) MRI systems (Ingenia 1.5T, Ingenia 3T, Achieva 3T: Philips Healthcare, Eindhoven; MAGNETOM Vida 3T: Siemens, Erlangen) with a 32-channel head coil. MRI protocols are summarized in Table 1. For DSC-MRI, a 15-mL intravenous bolus of gadobenate dimeglumine (Multihance, Bracco diagnostics, Singen, Germany) or gadoteridol (ProHance, Bracco diagnostics) was administered using a power injector through a peripheral arm vein at a flow rate of 5.0 mL/s, followed by a 20-mL saline flush. An additional 5 mL of contrast agent was administered 5 minutes prior to the dynamic perfusion imaging. Pediatric patients received 2 mL/kg of total contrast material (ProHance). The parameters of fast field echo T2*-weighted imaging were as follows: plane, axial; repetitive time, 1500 to 1840 ms; echo time, 30 to 50 ms; number of excitations, 1; slice thickness, 4 to 5 mm; slice increment, 5 to 5.2 mm; field of view, 226 to 235 mm; matrix, 128 × 128 - 144 × 144; flip angle, 40 to 90 degree; dynamic measurements, 40–70.

**Table 1:** MRI acquisition protocol.

	T2WI	FLAIR	Pre-and post-contrast fat-sat T1WI	DWI (b = 0, 1000 s/mm^2^)	Fast field echo T2*WI
Plane	Axial	Axial	Axial	Axial	Axial
Repetitive time (ms)	3930–5906	8500–11000	500–2300	3529–5960	1500–1840
Echo time (ms)	80–110	105–140	5–20	58.2–91.2	30–50
Flip angle (degree)	90–135	90–150	69–125	90–180	40–90
Number of excitations	1–3	1, 2	1,2	1,2	1
Slice thickness/increment (mm)	4–5/4.4–6	4–5/4.4–6	4–5/4.4–6	4–5/4.4–5	4–5/5–5.2
Field of view (mm)	227–236	228–252	160–240	227–251	226–235
Matrix	224 × 224–560 × 560	320 × 310–560 × 560	188 × 188–320 × 320	176 × 176–320 × 320	128 × 128–144 × 144

DWI = diffusion-weighted imaging, FLAIR = fluid-attenuated inversion recovery, MRI = magnetic resonance imaging, T1WI = T1-weighted imaging, T2*WI = T2*-weighted imaging, T2WI = T2-weighted imaging.

### 2.4. Conventional MRI analyses

Tumor size (maximum tumor diameter in axial slice), location (supratentorial or infratentorial), and shape based on contrast enhancement (solid, solid and cystic, or cystic) were analyzed by a board-certified radiologist with 6 years of experience in neuroradiology under the direct supervision of another board-certified radiologist with 13 years of experience in neuroradiology. The 2 radiologists were blinded to the tumor type. The margins of the combined area of the contrast-enhanced components and cystic components of the tumor were defined as the tumor margins.

### 2.5. Quantitative DSC-MRI analyses

Quantitative DSC-MRI analyses were conducted using OleaSphere (Version 3.0; Olea Medical, La Ciotat, France). DSC-MRI data were processed with motion artifact correction using rigid-body registration. The arterial input function (AIF) was calculated automatically using cluster analysis techniques. AIF deconvolution was performed with a time-insensitive block-circulant singular-value decomposition.^[21]^ Whole-brain relative CBV (rCBV) and relative CBF (rCBF) maps were generated using voxel-wise division of the area under the concentration-time curve by the area under AIF. Under the direct supervision of a board-certified radiologist with 13 years of experience in neuroradiology, a board-certified radiologist with 9 years of experience in neuroradiology carefully delineated regions-of-interest (ROIs) by freehand on every axial slice of perfusion maps depicting tumors to generate volumes-of-interest (VOIs). Both radiologists were blinded to the pathological diagnoses of the tumors. Cystic, necrotic, or hemorrhagic regions and vessels were carefully excluded from the ROIs for analyses using reference T2-weighted, FLAIR, pre-and post-contrast T1-weighted, and T2*-weighted images. Another ROI was placed over normal-appearing supratentorial white matter as a reference to correct for age- and patient-dependent variations in perfusion parameters.^[12,22]^ VOIs and reference ROIs on perfusion maps were transposed to rCBV and rCBF maps. Normalized rCBV (nrCBV) and rCBF (nrCBF) were calculated by dividing the mean rCBV and rCBF of the tumor by those of the reference ROIs.

The percentage signal recovery (PSR) was calculated using the following formula^[23]^: PSR = 100% × [S1 − Smin]/ [S0 − Smin], where S0 was the baseline signal intensity averaged over the first 10 time points, S1 was the tail averaged over the last 10 time points, and Smin was the minimum T2*-weighted signal intensity in the dynamic series. To calculate PSR, a circular ROI (30–40 mm^2^) was placed within solid components of the tumors.

### 2.6. Quantitative ADC analyses

DWI was performed for all patients. ADC maps were generated using OleaSphere. VOIs were generated for solid components of the tumors as described for DSC-MRI analysis. The reference ROI was placed in normal-appearing white matter, and the normalized mean ADC (nADCmean) was calculated for each tumor VOI by dividing ADC values by the mean ADC of the reference ROIs.^[24]^

### 2.7. Statistical analysis

Tumor size, DSC-MRI parameters (nrCBV, nrCBF, and PSR), and nADCmean were compared between the 3 tumor groups using the Kruskal-Wallis and post hoc Mann-Whitney *U* tests. Tumor location and shape were compared between the 3 groups using Fisher’s exact tests. The area under the receiver operator characteristic curve (AUC) of each MRI parameter was calculated using the optimal threshold values determined by the highest Youden index (sensitivity + specificity − 1).^[25]^ Family-wise error was corrected using the Bonferroni method. Family-wise error-corrected 2-sided *P* values of <.05 were considered statistically significant. All statistical analyses were performed using R software (version 4.1.1; R Foundation for Statistical Computing, Vienna, Austria).

## 3. Results

### 3.1. Patients

The demographic data of the study population are summarized in Table 2. This study included 23 (median age, 7 years [range, 2–26 years]; 12 female), 13 (10 years [1–24 years]; 3 females), and 12 (43 years [23–73 years]; 7 females) patients with PA, MB, and HB, respectively. Eleven out of 12 PAs showed KIAA-BRAF fusion, other 2 PAs showed neither BRAF V500E/K mutation, while molecular feature of the other 9 PAs were unknown. Four, 1, 2, and 6 MBs were WNT-activated, SHH-activated and TP53-wildtype, SHH activated with unknown TP53 status, and non-WNT/non-SHH-activated, respectively. Four patients with HB were diagnosed with von Hippel-Lindau disease.

**Table 2:** Patient demographic data.

	Pilocytic astrocytoma	Medulloblastoma	Hemangioblastoma
Number of patients	23	13	12
Age (median, yrs [range])	7 [2–26]	10 [1–24]	43 [23–73]
Sex (Male: Female)	11:12	10:3	4:7

### 3.2. Imaging findings

**Figure 1.**
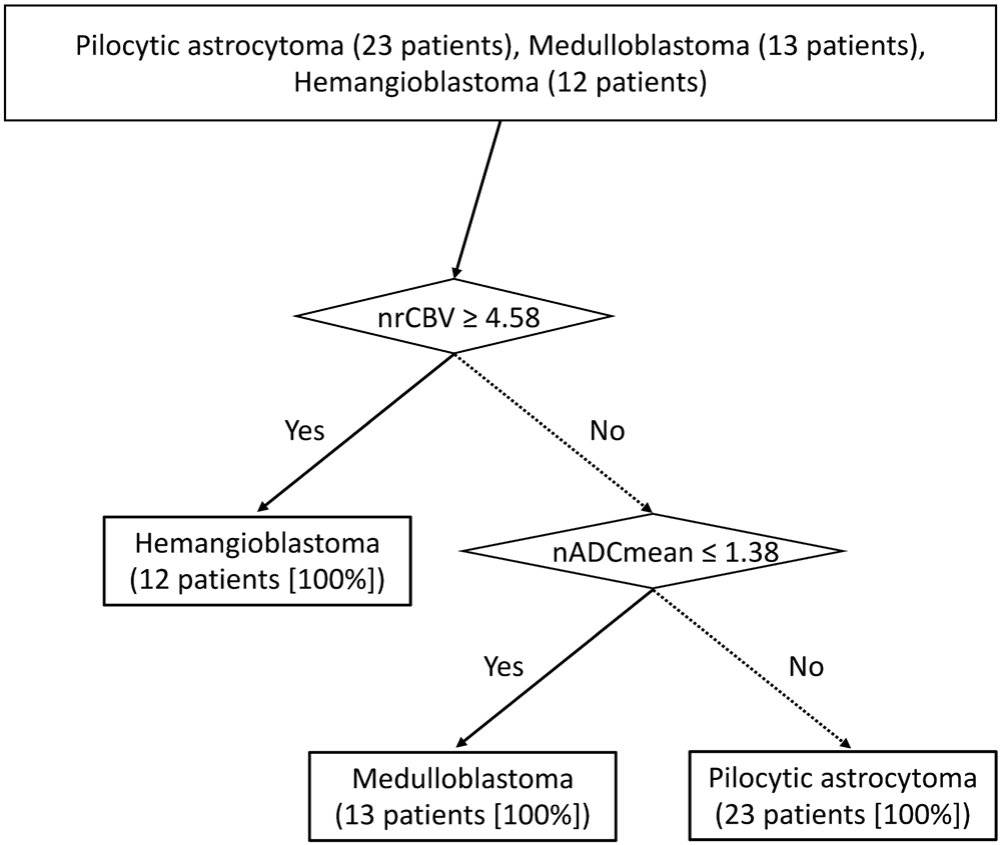
An algorithmic approach to differentiate pilocytic astrocytoma, medulloblastoma, and hemangioblastoma. Using a cutoff value of 4.58 for normalized relative cerebral blood volume (nrCBV), all hemangioblastomas were diagnosed. Subsequently, using a cutoff value of 1.38 for nADC mean, all medulloblastomas and pilocytic astrocytomas were diagnosed.

**Figure 2.**
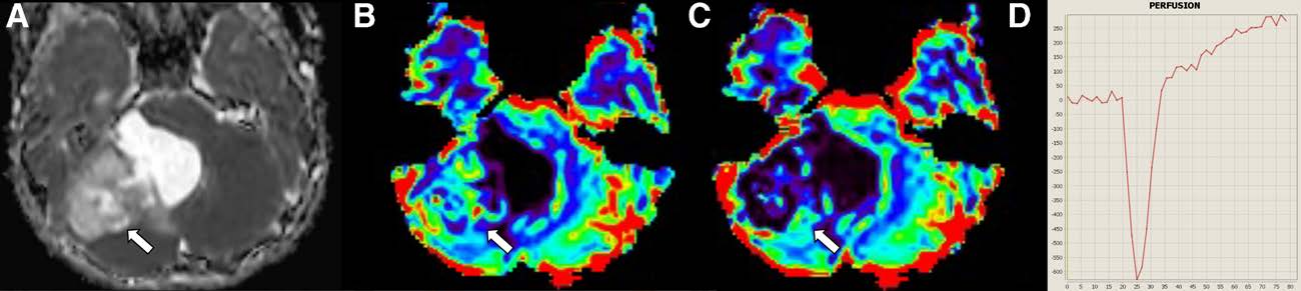
Pilocytic astrocytoma in an 11-year-old boy. MRI shows a solid cystic mass in the right cerebellar hemisphere. Normalized mean apparent diffusion coefficient is 1.94 (a, arrow). Normalized relative cerebral blood flow and volume are 2.07 and 2.28, respectively (b and c, arrows). The percentage signal recovery is 141.6 (d). MRI = magnetic resonance imaging.

**Figure 3.**
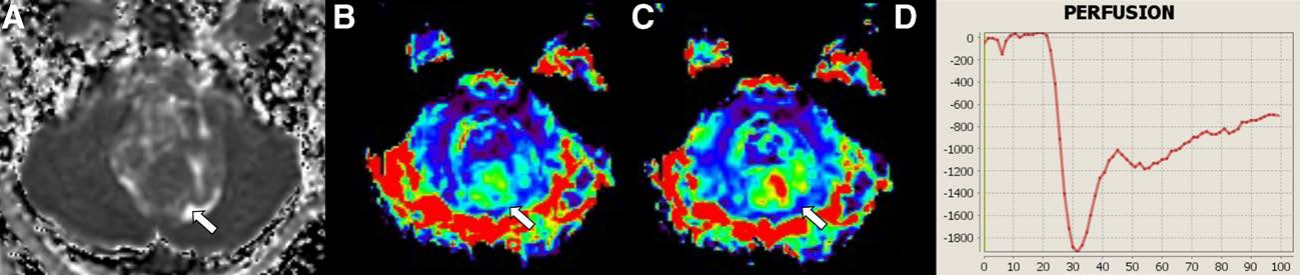
Medulloblastoma in a 21-year-old man. MRI shows a lobulated mass near the fourth ventricle. Normalized mean apparent diffusion coefficient is 1.37 (a, arrow). Normalized relative cerebral blood flow and volume are 1.37 and 2.27, respectively (b and c, arrows). The percentage signal recovery is 62.2 (d). MRI = magnetic resonance imaging.

**Figure 4.**
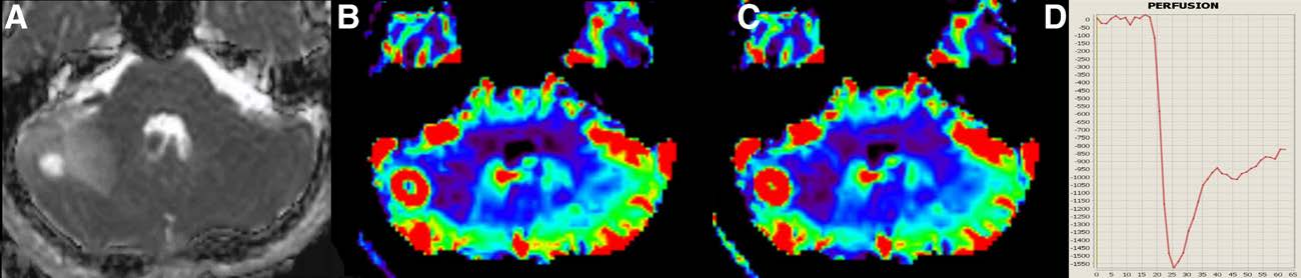
Hemangioblastoma in a 25-year-old man. MRI shows an oval mass in the right cerebellar hemisphere. Normalized mean apparent diffusion coefficient is 1.17 (a, arrow). Normalized relative cerebral blood flow and volume are 5.53 and 8.97, respectively (b and c, arrows). The percentage signal recovery is 43.0 (d). MRI = magnetic resonance imaging.

Imaging results are summarized in Tables 3 and 4. The size of HB was significantly smaller than PA or MB (PA: 49 mm [17–80 mm] vs MB: 39 mm [30–50 mm] vs HB: 27.5 mm [8–48 mm], *P* = .0054 for PA vs MB; *P* = .026 for MB vs HB). nADCmean was significantly higher in PA than in MB (PA: median, 2.2 [range, 1.59–2.65] vs MB: 0.93 [0.70–1.37], *P* < .001). nrCBF was significantly higher in HB than in PA and MB (PA: 1.10 [0.54–2.26] vs MB: 1.62 [0.93–3.16] vs HB: 7.83 [2.75–20.1], all *P* < .001). nrCBV differed significantly between all 3 tumor types (PA: 0.89 [0.34–2.28] vs MB: 1.69 [0.93–4.23] vs HB: 8.48 [4.59–16.3], *P* = .014 for PA vs MB; *P* < .001 for PA vs HB and MB vs HB). PSR was significantly lower in HB than in PA and MB (PA: 125.9 [53.7–1046.0] vs MB: 110.7 [62.2–327.4] vs HB: 53.8 [8.98–111.4], all *P* < .001). Using threshold values of 4.58 for nrCBV (HB > PA or MB; AUC, 1.0) and 1.38 for nADCmean (PA > MB; AUC, 1.0), all tumors were successfully differentiated from other tumor types (Fig. 1). Representative cases of PA, MB, and HB are presented in Figures 2, 3, and 4, respectively.

**Table 3:** Conventional MRI findings.

Parameters	Pilocytic astrocytoma (PA)	Medulloblastoma (MB)	Hemangioblastoma (HB)	Family-wise error-corrected *P* value			
Number of patients	23 patients	13 patients	12 patients	All patients	PA vs MB	PA vs HB	MB vs HB
Size (mm)	49 [17–80]	39 [30–50]	27.5 [8–48]	**0.0020** *	0.50	**0.0054** *	**0.026** *
Shape				0.061	NA		
Solid	5	10	2				
Solid and cystic	17	3	10				
Cystic	1	0	0				
Supratentorial: Infratentorial	6: 17	0: 13	0: 12	0.59	NA		

HB = hemangioblastoma, MB = medulloblastoma, MRI = magnetic resonance imaging, nADCmean = normalized mean apparent diffusion coefficient, nrCBF = normalized relative cerebral blood flow, nrCBV = normalized relative cerebral blood volume, PA = pilocytic astrocytoma, PSR = percentage signal recovery.

* Statistically significant.

**Table 4:** Dynamic susceptibility contrast perfusion MRI findings.

Parameters	Pilocytic astrocytoma (PA)	Medulloblastoma (MB)	Hemangioblastoma (HB)		Family-wise error-corrected *P* value		
Number of patients	23 patients	13 patients	12 patients	All patients	PA vs MB	PA vs HB	MB vs HB
nADCmean	2.2 [1.59–2.65]	0.93 [0.70–1.37]	1.52 [0.65–3.0]	**<0.001** *	**<0.001** *	0.68	0.41
nrCBF	1.10 [0.54–2.26]	1.62 [0.93–3.16]	7.83 [2.75–20.1]	**<0.001** *	0.1	**<0.001** *	**<0.001** *
nrCBV	0.89 [0.34–2.28]	1.69 [0.93–4.23]	8.48 [4.59–16.3]	**<0.001** *	**0.014** *	**<0.001** *	**<0.001** *
PSR	125.9 [53.7–1046.0]	110.7 [62.2–327.4]	53.8 [8.98–111.4]	**<0.001** *	>0.99	**<0.001** *	**<0.001** *

HB = hemangioblastoma, MB = medulloblastoma, MRI = magnetic resonance imaging, nADCmean = normalized mean apparent diffusion coefficient, nrCBF = normalized relative cerebral blood flow, nrCBV = normalized relative cerebral blood volume, PA = pilocytic astrocytoma, PSR = percentage signal recovery.

* Statistically significant.

## 4. Discussion

This study compared conventional and advanced MRI findings including DSC-MRI parameters and nADCmean between PA, MB, and HB using VOI analyses. Significant differences were observed in all DSC-MRI parameters and nADCmean when comparing any 1 or more combinations. Notably, all tumors were successfully differentiated using an algorithmic approach with a threshold value of 4.58 for nrCBV and subsequent threshold value of 1.38 for nADCmean.

Various ADC parameters (e.g., mean, minimum, variance, skewness, kurtosis, or percentiles) have been reported to be useful for differentiating PA and MB, but the measurement methods (i.e., subjective signal intensity or objective values derived from 2-dimensional ROIs or 3-dimensional VOIs) used in previous studies were inhomogeneous^.[7–12]^ Nevertheless, reports consistently indicate that ADC values are lower in MB than in PA. For example, Phuttharak et al reported an AUC of 0.9936 for differentiating MB from PA using a cutoff nADCmean of ≤ 1.17,^[6]^ which agrees with our findings. In contrast, other studies have failed to differentiate PA and HB using ADC.^[16,17]^ The difference in ADC values in PAs and MBs can be explained by their histological features; whereby PAs contain abundant myxoid stroma and MBs comprise densely packed tumor cells.^[1,26]^

She et al reported that relative peak height and PSR for DSC-MRI were higher in HB than in PA. Notably, threshold values of ≥ 3.2 for relative peak height and ≤ 0.9 for relative PSR differentiated HB from PA with accuracies of 91.7% and 83.3%, respectively.^[2]^ However, the DSC-MRI parameters most frequently used in routine clinical practice (rCBV and rCBF) were not examined in their study.^[2]^ Neska-Matuszewska et al reported that a threshold value of ≥ 3.74 for rCBV differentiated HB from PA without overlap, but the number of included cases was limited.^[16]^ The distinctly higher nrCBV and nrCBF in HB than in PA observed herein may be attributable to the prominent capillary networks in HB.^[4]^ In this regard, the significantly lower PSR in HB than in PA implies greater capillary permeability in HB, thereby permitting PSR to stay suppressed due to the T2* effects of the contrast material in the extravascular extracellular space.^[27]^

Knowledge of the differences in DSC parameters between PA and MB is limited. Kurokawa et al compared nrCBV and nrCBF between PA and MB and reported that both parameters were significantly higher in MB than in PA, but overlapping values were also observed.^[12]^ By harnessing an algorithm that applied nrCBV and nADCmean thresholds in this order, we accurately differentiated all 3 tumors without overlap (Fig. 1). We believe that this approach is more applicable in daily clinical practice, whereby differentiation of only 2 tumor types is generally insufficient.

This study has several limitations. First, this was a single-institution retrospective study. Second, the number of included cases was limited. Third, MRI protocols were not homogeneous owing to differences in machines and vendors. However, we mitigated the risk of heterogeneity of MRI parameters by normalization. Fourth, the age range of the study population was wide, but these tumors occur in both children and adults. Further studies with larger study populations are warranted to validate the effectiveness of the algorithmic approach proposed in this study.

## 5. Conclusion

DSC parameters and nADCmean were significantly different in comparisons of any 1 or more combinations of PA, MB, and HB. An algorithmic approach combining nrCBV and nADCmean may be useful for differentiating these tumor types.

## Acknowledgments

We would like to thank Editage [http://www.editage.com] for editing and reviewing this manuscript for English language.

## Author contributions

**Conceptualization:** Ryo Kurokawa.

**Data curation:** Ryo Kurokawa, Akira Baba.

**Formal analysis:** Ryo Kurokawa.

**Investigation:** Ryo Kurokawa.

**Methodology:** Ryo Kurokawa, Mariko Kurokawa.

**Supervision:** Toshio Moritani.

**Validation:** Toshio Moritani.

**Visualization:** Ryo Kurokawa.

**Writing – original draft:** Ryo Kurokawa.

**Writing – review & editing:** Mariko Kurokawa, Akira Baba, John Kim, Aristides Capizzano, Jayapalli Bapuraj, Ashok Srinivasan, Toshio Moritani.
